# Impact of the new heart allocation policy on patients with restrictive, hypertrophic, or congenital cardiomyopathies

**DOI:** 10.1371/journal.pone.0247789

**Published:** 2021-03-02

**Authors:** Fouad Chouairi, Clancy W. Mullan, Sounok Sen, Makoto Mori, Michael Fuery, Robert W. Elder, Joshua Lesse, Kelsey Norton, Katherine A. Clark, P. Elliott Miller, David Mulligan, Richard Formica, Joseph G. Rogers, Daniel Jacoby, Christopher Maulion, Muhammad Anwer, Arnar Geirsson, Nihar R. Desai, Tariq Ahmad

**Affiliations:** 1 Yale School of Medicine, New Haven, Connecticut, United States of America; 2 Division of Cardiac Surgery, Yale School of Medicine, New Haven, Connecticut, United States of America; 3 Section of Cardiovascular Medicine, Yale School of Medicine, New Haven, Connecticut, United States of America; 4 Section of Pediatric Cardiology, Yale School of Medicine, New Haven, Connecticut, United States of America; 5 Division of Transplantation, Yale School of Medicine, New Haven, Connecticut, United States of America; 6 Section of Nephrology, Yale School of Medicine, New Haven, Connecticut, United States of America; 7 Division of Cardiology, Duke University Medical Center, Durham, North Carolina, United States of America; Ohio State University Wexner Medical Center Department of Surgery, UNITED STATES

## Abstract

**Background:**

Patients with restrictive or hypertrophic cardiomyopathy (RCM/HCM) and congenital heart disease (CHD) do not derive clinical benefit from inotropes and mechanical circulatory support. Concerns were expressed that the new heart allocation system implemented in October 2018 would disadvantage these patients. This paper aimed to examine the impact of the new adult heart allocation system on transplantation and outcomes among patients with RCM/HCM/CHD.

**Methods:**

We identified adult patients with RCM/HCM/CHD in the United Network for Organ Sharing (UNOS) database who were listed for or received a cardiac transplant from April 2017-June 2020. The cohort was separated into those listed before and after allocation system changes. Demographics and recipient characteristics, donor characteristics, waitlist survival, and post-transplantation outcomes were analyzed.

**Results:**

The number of patients listed for RCM/HCM/CHD increased after the allocation system change from 429 to 517. Prior to the change, the majority RCM/HCM/CHD patients were Status 1A at time of transplantation; afterwards, most were Status 2. Wait times decreased significantly for all: RCM (41 days vs 27 days; P<0.05), HCM (55 days vs 38 days; P<0.05), CHD (81 days vs 49 days; P<0.05). Distance traveled increased for all: RCM (76 mi. vs 261 mi, P<0.001), HCM (88 mi. vs 231 mi. P<0.001), CHD (114 mi vs 199 mi, P<0.05). Rates of transplantation were higher for RCM and CHD (P<0.01), whereas post-transplant survival remained unchanged.

**Conclusions:**

The new allocation system has had a positive impact on time to transplantation of patients with RCM, HCM, and CHD without negatively influencing survival.

## Introduction

It has long been recognized that symptomatically advanced heart failure patients with restrictive or hypertrophic cardiomyopathy (RCM/HCM) and congenital heart disease (CHD) are at high risk for adverse outcomes but are not candidates for conventional therapies that benefit patients with left ventricular systolic dysfunction [1–3]. Intravenous inotropes and temporary mechanical support tend to be ineffective and poorly tolerated, making it difficult for these patients to meet conventional criteria required for an appropriate urgency status on the transplant list. Furthermore, these patients generally do not qualify for durable left ventricular assist devices [4, 5].

In 2018, the Organ Procurement and Transplantation Network (OPTN) allocation system modified adult heart allocation by changing from a 3-tiered system to a 6-tiered system [6]. Prior to the change, patients with HCM, RCM, and CHD were qualified as status 2, a low status, and had limited options to attain a higher tier status without submission of exception requests. The new allocation system took this into account and allowed for a higher listing status for these patients (status 4, patients who require a transplant but can be discharged home). Since the policy change continued to prioritize patients with cardiogenic shock needing mechanical circulatory support that might lead to inequality among HCM/RCM/CHD patients several specific objective criteria were also introduced that would allow for higher urgency statuses for patients who met specific clinical and/or hemodynamic variables [7–9].

The impact of these changes on patterns of transplantation among HCM/RCM/CHD patients awaiting cardiac transplantation are unknown. To address these questions, we analyzed the United Network for Organ Sharing (UNOS) database from before and after the implementation of the new adult heart allocation system.

## Methods

### Data source

The Standard Transplant Analysis and Research (STAR) files with deidentified recipient and donor information were obtained from UNOS. This is a prospectively maintained registry of patients listed for and undergoing solid organ transplantation. Investigators with access to the data signed data use agreements with UNOS. This study was deemed exempt by the Yale School of Medicine Institutional Review Board.

### Study population

In order to include the same time period before and after the allocation system change, we queried the UNOS registry for all adult patients (age >18 years) who were listed for or received a cardiac transplant from April 2017 to June 2020, had a minimum 30 days of follow up time, and a diagnosis of RCM, HCM, and CHD. RCM included patients with restrictive amyloidosis, endocardial fibrosis, sarcoidosis, restrictive radiation/chemotherapy, and idiopathic restrictive myopathy. Patients with total artificial hearts, right ventricular assist devices, and multiorgan transplants were excluded from analysis. Patients with an initial listing before the allocation change and an end listing was after the allocation change were also excluded. The groups were further stratified into “Pre” and “Post” cohorts based on the allocation system methodology of patients: those listed before October 18, 2018 were the “Pre” whereas those after were the “Post” cohort.

### Statistical analysis

Continuous variables were presented as median with interquartile range using Mann-Whitney-Wilcoxon tests. Non-parametric testing was required due to skewed variable distributions. Categorical variables were described as percentage of count and compared with chi-squared tests. Survival analysis was performed using Kaplan-Meier estimates and the log-rank test for survival to compare patients listed before and after the allocation change. Patients who were transplanted and those removed from the waitlist due to recovery were censored. Unadjusted and adjusted Cox models were used to evaluate the association of listing era (Pre vs Post allocation change) with waitlist mortality, transplantation, post-transplantation death, and post-transplantation graft failure. Competing risks analyses were performed for waitlist survival, death, transplantation, and removal from waitlist due to recovery. Curves from each era were compared with Gray’s tests of inequality. Patients are followed per their transplant center’s post-transplantation policy and data is reported to UNOS accordingly. Death dates are supplemented using the Social Security Administration’s death master file on a monthly basis. Restricted cubic splines were generated for distance traveled from donor to recipient site. Statistical significance was considered at two-tailed P<0.05. All analyses were performed in SAS 9.4 (SAS Institute Inc, Cary, NC). Figures were produced in GraphPad Prism 8.3.0 (GraphPad Software, La Jolla, CA).

## Results

The baseline characteristics of patients at time of listing for cardiac transplantation during the study period are demonstrated in **Table 1**. 398 patients were listed with a diagnosis of RCM (182/216; pre/post), 238 patients were listed with a diagnosis of HCM (119/119 pre/post), and 310 were listed with a diagnosis of CHD (128/182 pre/post). With the allocations system change, the relative number of RCM patients and HCM patients did not increase significantly (RCM: 4.2% to 4.7%, P = 0.275, HCM: 3.1% to 2.9%, P = 0.541), while the relative number of CHD patients increased between the time periods (CHD: 3.3% to 4.4%, P = 0.015). In terms of transplantation, in the pre-allocation system changes time period there were 3030 total transplants (RCM: 125, HCM:106, CHD: 94) and in the post-allocation system changes time period there were 3488 total transplantations (RCM: 167, HCM: 107, CHD: 140). Patient characteristics were similar in either time period.

**Table 1:** Baseline characteristics of candidates at time of listing.

	Restrictive Cardiomyopathy			Hypertrophic Cardiomyopathy			Congenital Heart Disease		
Variables	Pre (N = 182)	Post (N = 216)	P	Pre (N = 119)	Post (N = 119)	P	Pre (N = 128)	Post (N = 182)	P
Age	61 (53–67)	60 (48–66)	0.299	50 (41–62)	53 (42–59)	0.979	36.0 (23.5–45.0)	38.0 (25.0–49.0)	0.195
Female	25.8	31.5	0.215	44.5	42.0	0.695	45.3	42.9	0.668
BMI	25.9 (23.6–29.3)	26.5 (23.1–30.2)	0.618	27.5 (23.4–31.8)	28.5 (24.0–32.8)	0.207	24.1 (21.1–28.5)	25.3 (21.0–29.5)	0.563
**Race/Ethnicity**			0.729			0.318			0.435
White	61.5	57.9		72.3	75.6		75.0	69.8	
Black	30.8	31.9		13.5	10.9		6.3	12.1	
Hispanic	4.4	6.9		6.7	8.4		12.5	12.6	
Asian	2.2	2.8		4.2	5.0		3.9	4.4	
**Pre-Transplant Support**									
Ventilator	1.1	2.3	0.358	2.5	0.8	0.313	3.9	2.2	0.378
Inotropes	35.2	37.0	0.699	26.9	21.9	0.365	37.5	35.2	0.673
LVAD	5.0	4.6	0.883	6.7	1.7	0.053	3.1	4.4	0.568
ECMO	1.1	3.2	0.152	4.2	2.5	0.472	3.1	2.2	0.612
IABP	5.5	14.8	0.003	2.5	6.7	0.123	1.6	7.7	**0.016**
**Comorbidities**									
Diabetes	15.4	18.5	0.408	8.4	16.8	0.051	7.8	10.4	0.434
ESRD	2.2	4.6	0.190	1.7	0.0	0.156	2.3	1.1	0.392
Tobacco user	35.2	28.7	0.167	36.1	40.3	0.505	23.4	21.1	0.628
Prior CVA	3.9	5.1	0.551	10.1	2.5	**0.016**	4.7	9.5	0.119
ICD	52.2	51.9	0.945	81.5	80.7	0.869	46.1	48.0	0.743
Prior Cardiac Surgery	9.9	9.3	0.831	24.4	28.6	0.463	82.7	78.4	0.358

**Fig 1:**
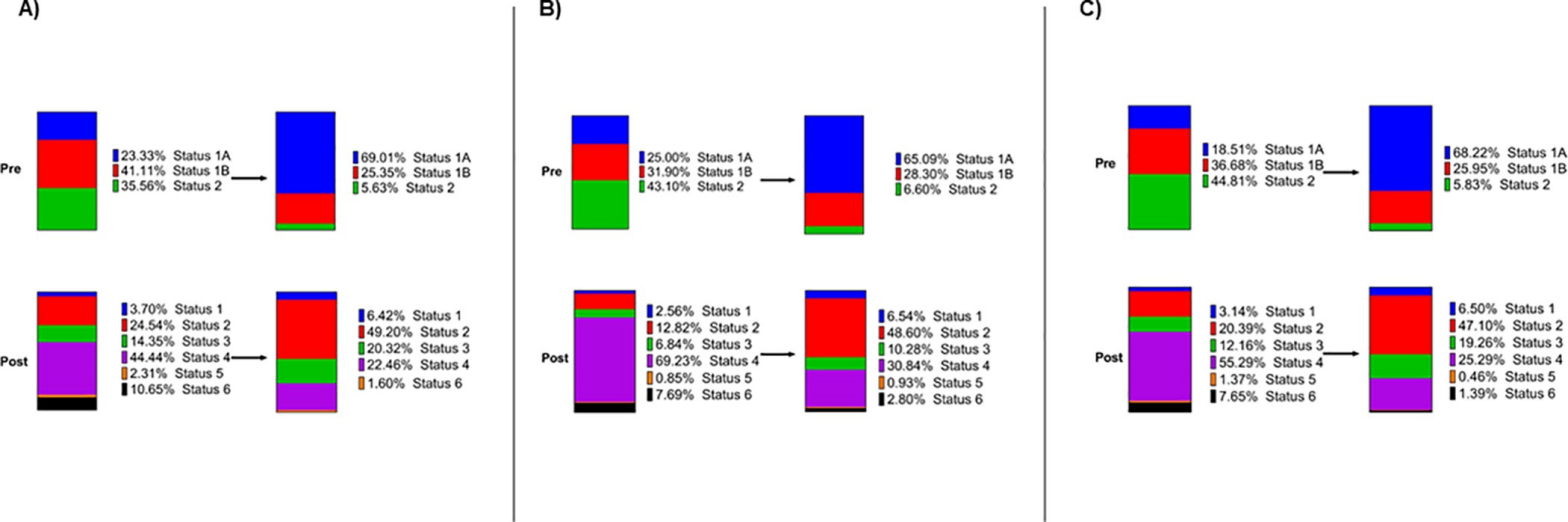
**Medical urgency status at time of listing (Left) and time of transplantation (Right) for before (Top) and after (Bottom) 2018 allocation system change.** Data is displayed for (A) RCM, (B) HCM, (C) CHD before and after implementation of new heart allocation system.

**S1 Fig** shows breakdown of listings according to OPTN region in the pre and post allocation change periods and trends across regions were generally unchanged. **Fig 1** demonstrates the medical urgency status at time of listing for RCM, HCM, and CHD in the pre and post eras. As shown, for RCM, the majority of patients were listed as UNOS Status 1B but were UNOS Status 1A at time of transplant. After the change in allocation system, the majority of patients were UNOS Status 4 at time of listing and Status 2 at time of transplant. In the case of HCM, the majority of patients were UNOS Status 2 at time of listing and UNOS Status 1A at time of transplant prior to the allocation system change. In the post period, the majority were listed as Status 4 but were Status 2 at time of transplant. Finally, for CHD, the majority of patients were UNOS Status 2 at time of listing and UNOS Status 1A at time of transplant prior to the allocation system change. In the post period, the majority were listed as Status 4 but were Status 2 at time of transplant. Wait times changed significantly for RCM, HCM, and CHD. In the case of RCM, the median days on the waitlist decreased from 41 (18–111) → 27 (8–75), P = 0.009. For HCM, the median days decreased from 55 (30–118) → 38 (12–94), P = 0.011. For CHD, the median days decreased from 81 (36–166) → 49 (18–118), P = 0.001.

**Fig 2:**
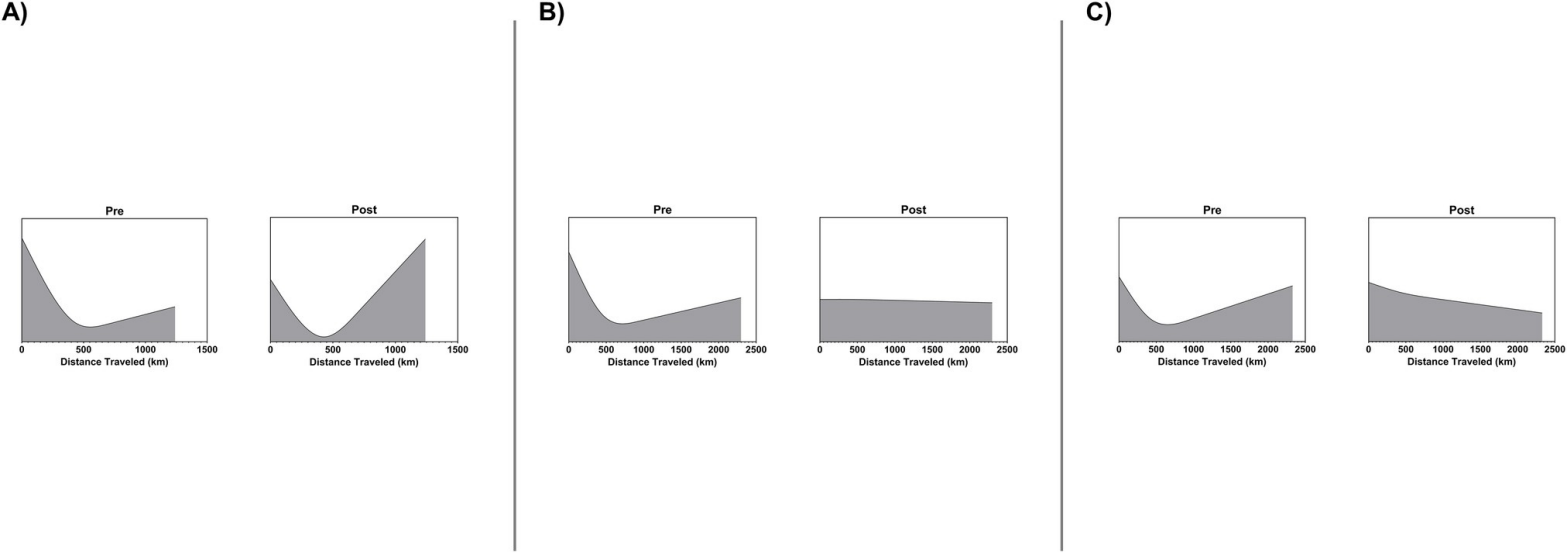
Distance traveled for organ retrieval by allocation system. Data is displayed for (A) RCM, (B) HCM, (C) CHD before and after implementation of new heart allocation system.

The baseline characteristics of donors is shown in **Table 2**. In general, characteristics for RCM, HCM, and CHD donors were similar in the prior and post periods. The key difference were longer ischemic times and distant traveled to procure the heart in the post allocation change period (**Fig 2**). There was an increase in the distance from donor to recipient hospital among all transplanted patients from 135→367 kilometers (P<0.001). In the case of RCM, median ischemic time increased from 3.0→3.5 hours and distant traveled increased from 122→420 kilometers (P<0.001, both). For HCM both ischemic time (3.0→3.3 hours) and distance traveled (142→ 370 kilometers) increased with borderline statistical significance (P = 0.058) for the former increase and P<0.001 for the latter. Finally, for CHD, there was no significant difference in ischemic time, but distance traveled increased from 183→320 kilometers (P = 0.032).

**Table 2:** Baseline characteristics of donors.

	Restrictive Cardiomyopathy			Hypertrophic Cardiomyopathy			Congenital Heart Disease		
Variables	Pre (N = 142)	Post (N = 187)	P	Pre (N = 106)	Post (N = 107)		Pre (N = 94)	Post (N = 140)	
Age (median IQR)	31 (25–41)	33 (25–39)	0.704	29 (23–40)	31 (24–41)	0.447	25 (22–32)	30 (23–36)	**0.008**
Female	32.4	34.8	0.653	37.7	34.6	0.632	42.6	35.7	0.292
BMI (median IQR)	25.9 (23.2–30.0)	27.3 (23.4–32.0)	0.107	25.8 (22.6–29.7)	26.4 (23.8–31.3)	**0.044**	24.5 (21.1–28.9)	26.0 (23.1–29.2)	0.110
High Risk Donor	32.4	36.4	0.454	30.2	29.9	0.964	28.7	33.6	0.434
Circulatory Death	0.0	0.5	0.383	0.0	1.9	0.157	0.0	2.1	0.276
**Race/Ethnicity**			0.972			0.509			0.117
White	60.6	63.1		68.9	68.2		63.8	60.7	
Black	14.8	12.8		13.2	14.0		22.3	14.3	
Hispanic	18.3	18.7		17.0	14.0		12.8	21.4	
**Substance Use**									
Alcohol Use	24.7	17.7	0.120	18.9	20.6	0.756	12.8	13.6	0.859
Tobacco user	10.6	11.2	0.848	6.6	19.6	**0.005**	3.2	6.4	0.271
Cocaine Use	26.1	25.1	0.849	26.4	30.8	0.475	17.0	15.0	0.678
Other Drug User	50.7	48.1	0.643	47.2	53.3	0.373	46.8	45.0	0.785
**Comorbidities**									
Hypertension	40.9	52.4	0.038	40.6	38.3	0.737	43.6	38.6	0.441
Diabetes	5.6	4.8	0.739	3.8	4.7	0.744	4.3	3.6	0.999
**Infections**									
Pneumonia	73.9	73.8	0.976	70.8	69.2	0.800	71.3	75.7	0.448
HCV	7.8	5.4	0.378	1.9	5.6	0.153	3.2	5.7	0.371
CMV	60.6	59.4	0.825	68.9	64.5	0.498	58.5	60.7	0.736
**Distance**									
Ischemic Time (hours) (median IQR)	3.0 (2.3–3.5)	3.5 (2.8–4.1)	**<0.001**	3.0 (2.3–3.6)	3.3 (2.6–3.8)	0.058	3.7 (2.8–4.3)	3.7 (3.0–4.3)	0.685
Distance Traveled (kilometers) (median IQR)	122 (23–365)	420 (126–668)	**<0.001**	142 (23–470)	370 (163–632)	**<0.001**	183 (32–539)	320 (84–592)	**0.032**

Key pre-operative support devices and post-operative events by allocation system are shown in **Table 3**. Notably, there was a significant increase in IABP support at time of transplant—more than two-fold—in the new allocation system for RCM, HCM, and CHD. There was also a significant decrease in the number of LVADs at time of transplant for patients with HCM. We noted no differences in post-operative adverse outcomes.

**Table 3:** Pre-transplant support and post-operative events.

	Restrictive Cardiomyopathy			Hypertrophic Cardiomyopathy			Congenital Heart Disease		
Variables	Pre (N = 142)	Post (N = 187)	P	Pre (N = 106)	Post (N = 107)	P	Pre (N = 94)	Post (N = 140)	
**Pre-Transplant Support**									
IABP	9.9	31.0	**<0.001**	9.4	25.2	**0.002**	6.4	15.7	**0.031**
Inotropes	54.9	44.4	0.058	39.6	32.7	0.294	56.4	48.6	0.241
Ventilator	0.7	2.1	0.292	1.9	2.8	0.659	3.2	2.9	0.999
LVAD	6.3	5.3	0.703	9.4	1.9	**0.017**	7.4	5.7	0.596
**Post-Operative Events**									
Stroke	4.9	1.6	0.082	4.7	2.8	0.463	4.3	5.7	0.620
Dialysis	14.8	16.0	0.756	19.8	14.0	0.259	22.3	22.1	0.972
Pacemaker Placement	1.4	3.7	0.198	2.8	1.9	0.643	3.2	1.4	0.361
Transfusion	9.9	8.6	0.684	12.3	6.5	0.152	13.8	9.3	0.278
**Acute Rejection Prior to Discharge**			0.107			**0.032**			0.169
Yes, treated with anti-rejection medications	8.0	4.0		16.0	5.0		10.6	16.5	
Yes, not treated with additional antirejection agent	5.6	11.3		3.8	6.0		14.9	8.3	
No Acute Rejection	86.4	84.7		80.2	89.0		74.5	75.2	

**Fig 3:**
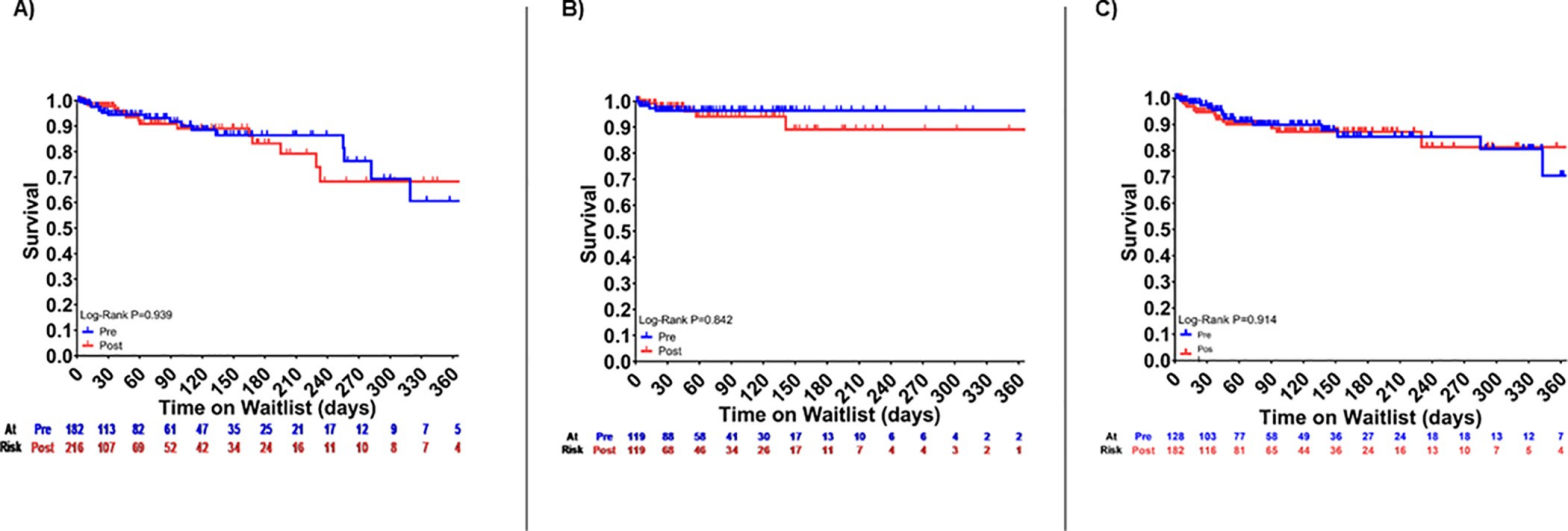
Unadjusted Kaplan-Meier curves for waitlist survival. Waitlist survival is displayed as (A) RCM, (B) HCM, (C) CHD before and after implementation of new heart allocation system.

**Fig 4:**
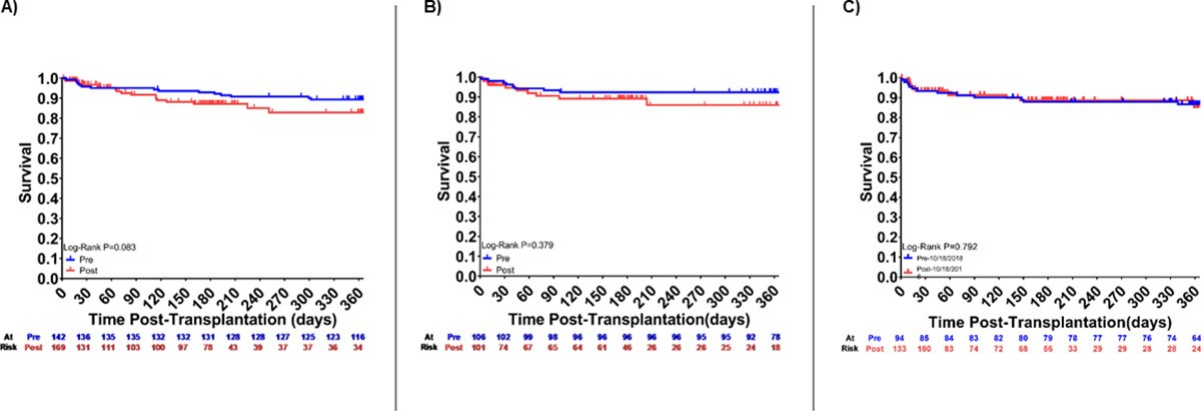
Post-transplant Kaplan-Meier survival curves. Post-transplant survival is displayed for (A) RCM, (B) HCM, (C) CHD before and after implementation of new heart allocation system.

**Fig 3** shows waitlist survival for RCM, HCM, and CHD prior to and after implementation of the new allocation system. As shown, there were no significant differences in survival on the waitlist with the change in allocation system. **S2 Fig** shows graft failure which has not changed significantly. **Fig 4** shows post-transplant survival for all causes of cardiomyopathy which has also not changed significantly.

**Fig 5:**
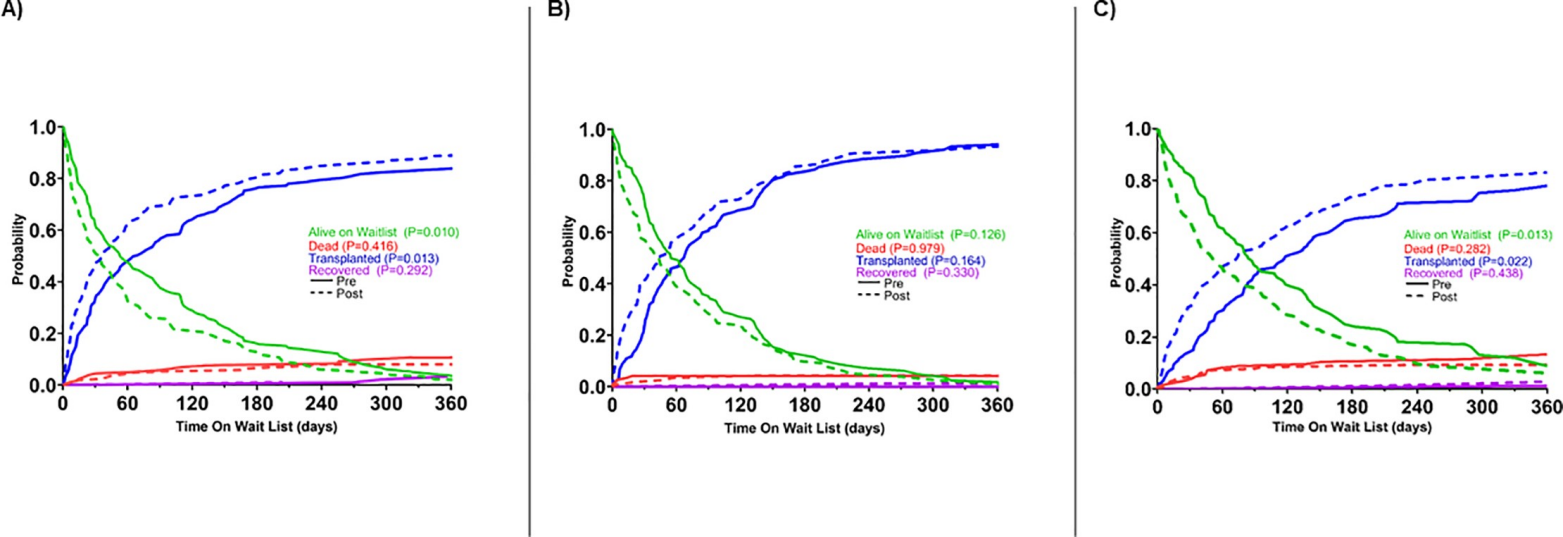
Competing risks for waitlist outcomes among patients listed for cardiac transplantation. Competing risks are displayed as A) RCM, (B) HCM, (C) CHD.

**Fig 5** demonstrates the competing risks for waitlist outcomes among patients listed for transplant with RCM, HCM, or CHD. In the case of RCM and CHD, there was a statistically significant increased likelihood of being transplanted with the new allocation system (P = 0.013 and P = 0.022). Whereas there was a similar trend in for HCM, it did not reach statistical significance (P = 0.16).

## Discussion

This study of the UNOS registry describes the impact of the 2018 change in the to the adult heart allocation system on transplantation of patients with restrictive and hypertrophic cardiomyopathy, and congenital heart disease (RCM, HCM, or CHD). We found that demographics of patients at time of listing were generally similar overall and across OPTN regions before and after implementation of the new allocation system. Whereas the majority of patients were at the Tier 4 urgency status at time of listing, they tended to be at Tier 2 urgency status at time of transplantation. This appears to be via use of intra-aortic balloon pumps (IABP) rather than disease specific status exceptions created for this patient population. Time for patients on the waitlist decreased significantly, while the use of donors from further distances increased. Rates of transplantation were significantly higher for RCM and CHD, with a trend in towards higher rates for HCM, whereas short term outcomes were unchanged. Overall, these data suggest that the new allocation system has motivated increased rates of transplantation for patients with RCM, HCM, and CHD—presumably via procurement of organs from greater distance—with no increase in short term adverse events.

Our findings are important for several reasons. First, concerns were raised that since the new allocation criteria were so heavily dependent on stratification based on interventions that do not generally benefit patients with RCM, HCM and CHD, these patients may have difficulty meeting criteria for higher status despite having a waitlist mortality equivalent to other candidates at higher statuses [10]. However, contrary to these concerns, time on the waitlist was significantly lower under the new allocation system, decreasing by more than 30%. Furthermore, there were no differences in outcomes post transplantation. This suggests that the allocation system changes resulted in better outcomes for this patient population.

Second, it appears that the greater rates of transplantation did not result from any significant differences in donor characteristics other than greater distances traveled to procure hearts. Whereas there has been controversy in terms of accepting donors from greater distances, it is broadly recognized that a majority of centers may be too conservative in their estimation of this risk, and our data shows that even a several fold increase in distance traveled did not result in worse outcomes [11, 12]. Therefore, it appears that RCM, HCM, and CHD patients benefited from one of the key changes to the allocation system that included broader sharing for the Status 1 and 2 patients (500-mile radius).

Third, we found that whereas the majority of patients were listed at Status 4, most were Status 2 at time of transplantation via use of intra-aortic balloon pumps (IABP), suggesting that this approach to improve prioritization for listing was being used rather than the standardized exceptions laid out by the OPTN (Organ Procurement and Transplantation Network) Review Board [13]. This indicates that centers might benefit from a greater consideration of the specific criteria for this patient population as a means to list at Status 2 urgency level when the clinical benefit from an IABP might be marginal. The increase in IABP use may be attributable primarily to the higher status afforded patients on IABPs rather than clinical benefit.

Finally, our competing risk analyses confirmed that the allocation system change has had a positive impact on patients with RCM, HCM, or CHD in a relatively short period of time. Despite this, it appears that some recent methods to increase the donor pool were not utilized. For example, only a minority of the donor hearts were hepatitis C nucleic acid testing (NAT) positive after the allocation system change, despite excellent outcomes with use of these organs [14]. Therefore, we believe that several avenues remain by which we can increase organ availability and decrease wait times for these patients as they await cardiac transplantation.

### Limitations

Despite utilizing the prospectively maintained UNOS registry, this analysis is limited by common issues with retrospective studies. Particularly, patients listed for transplant have been pre-selected by their listing institutions to be suitable transplant recipients, and these behaviors might have been modified in response to the allocation system change. Second, it is different to ascertain granular data about the clinical scenario that led to changes in listing of patients. Finally, we acknowledge that the allocation system has only been in effect for less than 2 years, and practices by the heart transplantation community are evolving [15, 16].

### Conclusions

This analysis of the UNOS database found that changes to the adult heart allocation system dramatically decreased time to transplantation for patients with RCM, HCM and CHD without negatively influencing survival. This appears to have been achieved via use of organs from greater distances. Only a minority of patients were Status 4 at time of transplantation, suggesting that they met the hemodynamic or end-organ dysfunction criteria for listing at a higher urgency status.

## Supporting information

S1 Fig Heart transplant candidates by region. Breakdown of Heart Transplant Candidates According to Organ Procurement and Transplantation Network (OPTN) Region Before and After Implementation of New Heart Allocation System.
(TIF)

S2 Fig Post-transplant graft failure Kaplan-Meier curves. Post-Transplant graft failure is displayed for (A) RCM, (B) HCM, (C) CHD Before and After Implementation of New Heart Allocation System.
(TIF)
