# Supplementary figures and images for: Impact of the new heart allocation policy on patients with restrictive, hypertrophic, or congenital cardiomyopathies
Source: PLoS One. 2021 Mar 2;16(3):e0247789. doi: 10.1371/journal.pone.0247789 (PMC7924739; doi:10.1371/journal.pone.0247789)

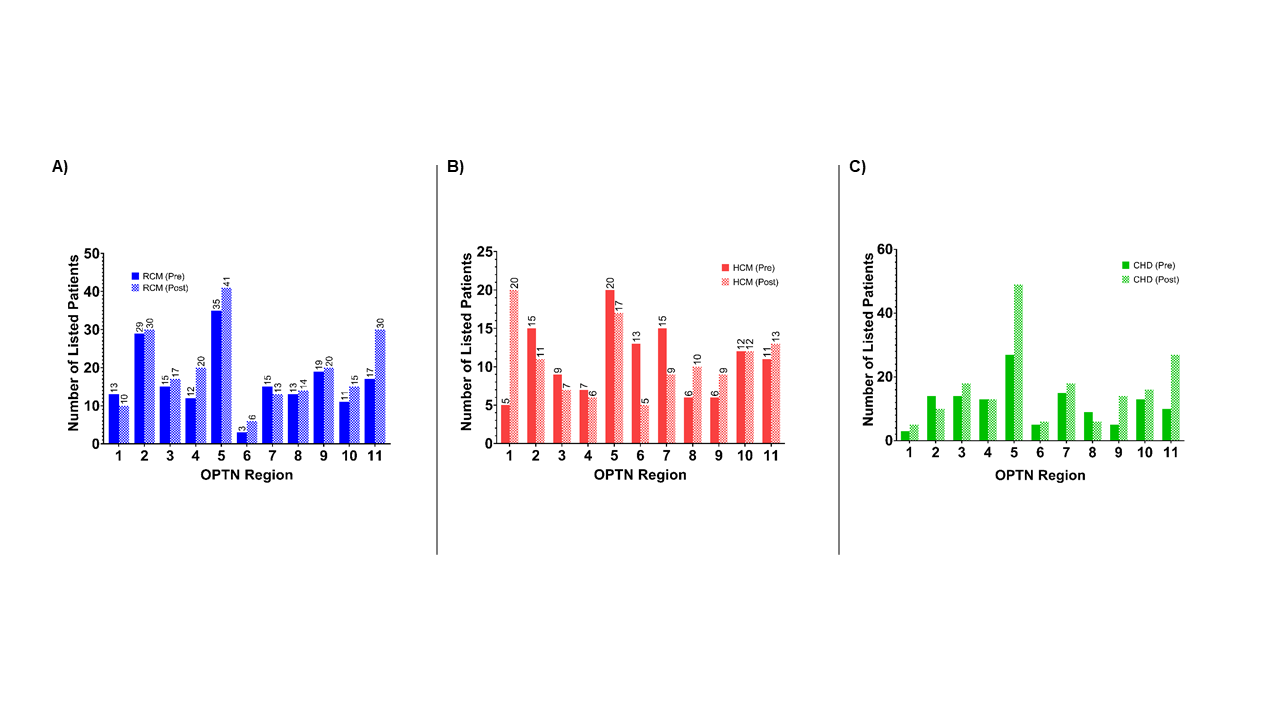

Supplement: S1 Fig — Breakdown of Heart Transplant Candidates According to Organ Procurement and Transplantation Network (OPTN) Region Before and After Implementation of New Heart Allocation System. (TIF) [file pone.0247789.s001.tif]

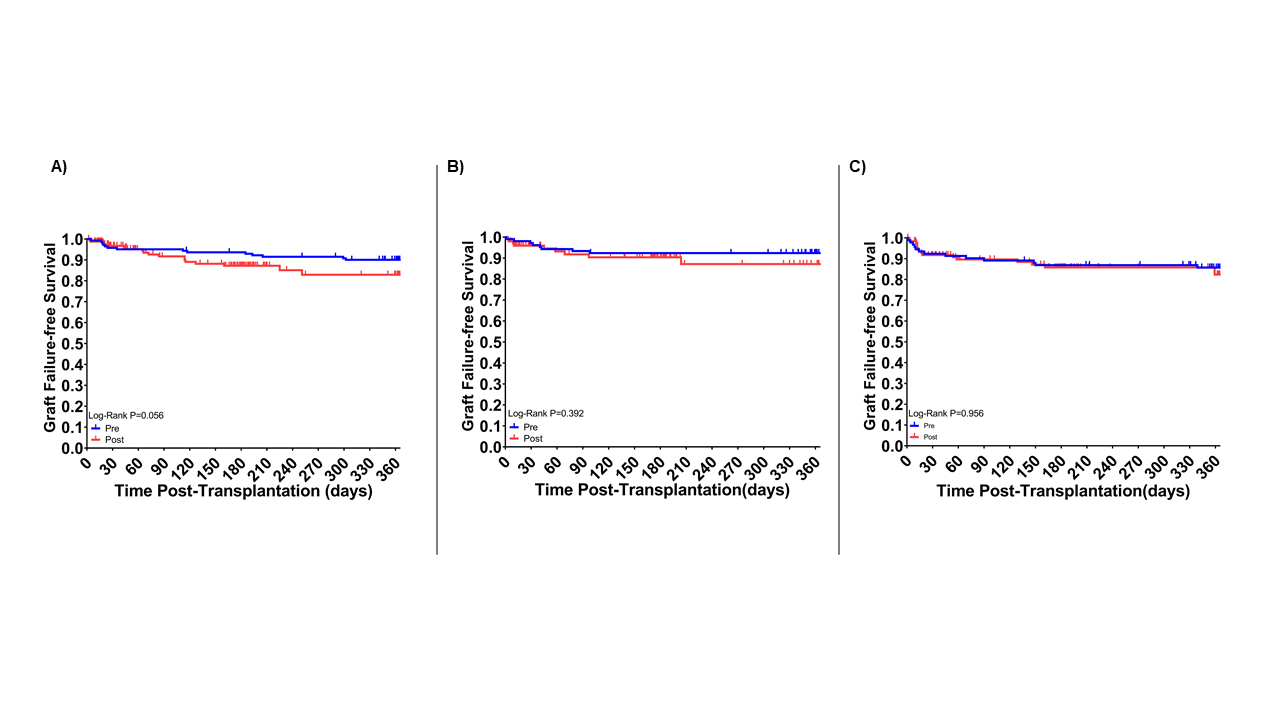

Supplement: S2 Fig — Post-Transplant graft failure is displayed for (A) RCM, (B) HCM, (C) CHD Before and After Implementation of New Heart Allocation System. (TIF) [file pone.0247789.s002.tif]
